# Editorial: The right heart: a key target for cardiovascular medicine

**DOI:** 10.3389/fcvm.2025.1713152

**Published:** 2025-10-24

**Authors:** Maria Concetta Pastore, Simona Sperlongano, Paolo Calabrò, Matteo Cameli

**Affiliations:** ^1^ Department of Medical Biotechnologies, Division of Cardiology, University of Siena, Siena, Italy; ^2^ Division of Cardiology, Department of Translational Medical Sciences, University of Campania Luigi Vanvitelli, Naples, Italy

**Keywords:** right heart, right ventricle (RV), heart failure, pulmonary hypertension, diagnosis, prognosis

**Editorial on the Research Topic**
The right heart: a key target for cardiovascular medicine

After being neglected for decades, the evaluation of the right ventricle (RV) has gained increasing interest among clinicians and researchers due to the strong evidence proving its importance for prognosis (1) in different clinical conditions, such as heart failure, valvular heart disease, and pulmonary hypertension (2–5). Although the RV is not the primary affected chamber in many pathological conditions, its involvement may be considered a marker of disease severity and transition to a poor prognosis (6). Moreover, new interventional techniques focused on the right heart and valves have been developed with significant improvement in quality of life (7).

This article Research Topic aimed at increasing scientific awareness of the right heart, highlighting the pivotal role of all its structures, which often must be considered not only as “collateral” but also as the main target for diagnostics, risk stratification, and therapy.

Research focusing on RV structural and functional impairment has led to the identification of new parameters to optimize the evaluation of this chamber, since its evaluation with common imaging modalities is sometimes limited (8). One of the emerging imaging modalities for the assessment of RV structure and function is 3D echocardiography (9), which offers a more comprehensive quantification of RV dimensions, with volumes comparable to cardiac magnetic resonance. This modality is gaining feasibility in parallel to the development of new automatic tools for the calculation of 3D parameters. Memis et al. achieved good results in the reproducibility of 3D echocardiography among expert and non-expert operators after a brief training to evaluate RV in patients with decompensated heart failure, which remains one of the main fields of application of RV assessment.

However, 3D echocardiography can sometimes be challenging in patients with pulmonary illness or who are poorly cooperative. Speckle tracking echocardiography (10–14) has been studied extensively and validated in multicenter studies to standardize acquisition and reference values (15). It offers a quick tool and sensitive parameters for RV function estimation, allowing for early diagnosis of subtle RV impairment and accurate prognostic assessment, particularly in primary and secondary pulmonary hypertension. Kaddoussi et al. showed how free-wall RV strain was useful in detecting subtle myocardial involvement in chronic obstructive pulmonary disease, despite normal basic echocardiographic parameters and left ventricular strain. The authors also found that RV strain was associated with a higher risk of hospitalization for acute exacerbation during the one-year follow-up (55% vs. 25% in patients with impaired vs. normal RV strain; *p* = 0.024). For these patients, the main target is to detect early signs of cor pulmonale in order to provide early therapy.

The same applies to patients with chronic thromboembolic pulmonary hypertension, a rare but severe cause of pulmonary hypertension (classified as “group 4”), which puts patients at high risk for decompensation and fatal events. Simeone et al., in two articles published in this Research Topic, provided an overview of the diagnostic algorithm and therapeutic strategies available for these patients, focusing on multidisciplinary evaluation and collaboration to optimize and personalize the treatment for this population.

Conversely, some authors claim that the use of imaging may be preceded by biomarkers for diagnosis. Cai et al. conducted research on rats undergoing surgical pulmonary artery banding with consequent RV remodeling to search for differentially expressed microRNAs (miRNAs), which were then validated in a cohort of 100 patients with either adaptive RV pressure overload (20), maladaptive RV pressure overload (20), left heart failure (20), or left ventricular hypertrophy (19) along with 21 controls. The authors identified circulating miRNA-486 as a marker of RV maladaptation in PH patients. This marker was not significantly different from B-type natriuretic peptide (BNP). It was also associated with the tricuspid annular plane systolic excursion/systolic pulmonary artery pressure (TAPSE/sPAP) ratio and BNP.

As previously mentioned, the RV plays a fundamental role in prognostic assessment in different clinical conditions, including cardiac surgery and left heart disease, since the pre-existence of RV dysfunction (16) or the development of considerable postoperative RV dysfunction is associated with a poor prognosis in patients undergoing cardiac surgery and may influence treatment choices. In fact, the scientific community is moving toward the research of reliable prognostic markers in these patients. Watanabe et al. proposed using QRS duration, as measured by a bipolar pacing catheter in the RV, as a perioperative monitoring parameter for RV function in patients undergoing robotic mitral valve surgery. The authors observed a prolongation of this parameter during robotic mitral valve repair, an association with right ventricular fractional area change (RVFAC), and they found that prolonged postoperative QRS_RV_ duration was the only significant parameter associated with a longer stay in the intensive care unit after surgery.

Finally, primary or acquired isolated right heart disease should not be overlooked, as it can have fatal consequences, albeit rarely. In particular, one of the main causes of cyanotic disease includes RV structure involvement, as depicted in the case report of Cui et al. on an infant with cyanosis and severe tricuspid regurgitation after high-altitude exposure due to spontaneous chordae tendineae rupture and tricuspid valve prolapse. The infant was initially misdiagnosed, but the case was promptly solved with cardiac surgery.

The field of diagnostic and therapeutic research on the right heart is continuously growing. It is hoped that this Research Topic will shed light on the importance of evaluating the right heart, provide new practical information for the management of these patients, and stimulate future research in this area.
